# Genetic polymorphism of long non-coding RNA TUG1 and susceptibility to polycystic ovary syndrome: a case-control study

**DOI:** 10.1186/s41065-026-00638-1

**Published:** 2026-01-22

**Authors:** Hua Zhang, Wei Liu, Xueqian Liu, Jingjing Ren, Yijiao Cheng, Yanjiao Liu, Yanjun Wu

**Affiliations:** 1 Department of Obstetrics and Gynecology, Liaocheng People’s Hospital, No.67 Dongchang West Road, Liaocheng, 252000 China; 2 Department of Obstetrics, Shenzhen Futian District Maternal and Child Health Hospital, Shenzhen, 518000 China; 3 Obstetrical Department, Affiliated Beijing Chao-Yang Hospital, Capital Medical University, No. 8, Gongti South Road, Chaoyang District, Beijing, 100020 China

**Keywords:** Polycystic ovary syndrome, SNP, TUG1, Genetic predisposition

## Abstract

**Background:**

Dysregulated expression of long non-coding RNA (lncRNA) TUG1 participates in the etiopathogenesis of polycystic ovary syndrome (PCOS). This study analyzed the genetic association of the TUG1 rs5749201 polymorphism in PCOS patients.

**Methods:**

Genotype and allele distributions of rs5749201 were analyzed in 210 PCOS patients and 230 healthy volunteers. Serum TUG1 levels were detected via qRT-PCR. The relationship between gene polymorphism and PCOS risk was analyzed via multivariate logistic regression.

**Results:**

Compared with the control group, the PCOS group had a significantly higher proportion of carriers with TUG1 rs5749201 AA genotype and a lower proportion of TT genotype carriers. Rs5749201 TT genotype carriers had notably reduced PCOS risk. This genetic association was also found in dominant and recessive models, with the locus independently linked to PCOS (OR = 0.427, 95%CI: 0.242–0.753; *P* = 0.003). AA genotype carriers had higher LDL, TG and FBS than AT/TT genotype carriers. PCOS patients had elevated TUG1 levels, with AA genotype carriers showing the highest.

**Conclusion:**

TUG1 rs5749201 was linked to PCOS susceptibility, which is correlated with its regulatory role in TUG1 expression.

**Supplementary Information:**

The online version contains supplementary material available at 10.1186/s41065-026-00638-1.

## Introduction

Polycystic ovary syndrome (PCOS) ranks among the most prevalent reproductive endocrine and metabolic disorders in females, impacting roughly 5%–10% of women of reproductive age. Its core features include ovulatory dysfunction, hyperandrogenemia, and polycystic ovarian morphology. Women with Polycystic Ovary Syndrome (PCOS) have a significantly increased risk of infertility, diabetes, dyslipidemia, hypertension, obesity and obstructive sleep apnea [1]. Studies have shown that the risk of metabolic syndrome in PCOS patients is four times higher than that in healthy women of the same age [2]. These metabolic irregularities can go on to trigger cardiovascular conditions, including myocardial ischemia, myocardial infarction, and acute coronary syndrome, complications that are severe and potentially fatal [3]. Although the pathogenesis of PCOS has not yet been fully elucidated, genetic factors are well-recognized as major contributors to its development. Among various genetic variations, single-nucleotide polymorphisms (SNPs) stand out as a key type that modulates an individual’s susceptibility to the condition.

SNPs, the most common type of genetic variation in the human genome, refer to changes in genomic DNA sequences where a single nucleotide is substituted, inserted, or deleted. Their ability to influence disease risk stems from their role in regulating gene expression or altering protein function. PCOS is a condition driven by both genetic and environmental factors, with genetic SNPs serving as a key genetic variation that dictates an individual’s vulnerability to the disorder [4, 5]. Through regulating the function of associated biological pathways, these SNPs indirectly raise the likelihood of PCOS onset. Existing literature strongly supports the significance of genetic factors in PCOS progression. Currently, polymorphic loci in multiple genes have been identified as promising biomarkers for the diagnosis and prognostic assessment of PCOS [6–8]. For example, polymorphisms in the vitamin D receptor (VDR) gene can alter the structural conformation and transcriptional activity of its encoded protein, thereby impairing vitamin D-mediated regulation of steroidogenesis in ovarian granulosa cells and glucose metabolism in pancreatic β-cells, and ultimately contributing to the pathogenesis of PCOS [9, 10].

In recent years, research on non-coding RNAs (ncRNAs) has significantly deepened insights into the pathogenesis of diverse diseases [11]. These molecules regulate gene expression at epigenetic, transcriptional, and post-transcriptional levels, and are pivotal for gene modulation during cell and embryonic development as well as the maintenance of organismal homeostasis. Pathway enrichment analysis integrated with hub gene-miRNA regulatory network construction has offered novel insights into identifying core regulatory elements and potential therapeutic targets in PCOS, while weighted gene co-expression network analysis (WGCNA) has emerged as a robust tool to discover novel regulatory long non-coding RNAs (lncRNAs), opening new avenues for dissecting the syndrome’s intricate regulatory networks [12, 13]. Notably, many lncRNAs interact closely with steroid hormone receptor function [14]. In PCOS patients, extensive abnormal lncRNA expression has been identified, pointing to their potential involvement in PCOS initiation and progression [15]. Additionally, investigations into the link between lncRNA gene polymorphisms and PCOS susceptibility are prevalent [16]. For example, Tan et al. reported an association between the rs10463297 SNP in the lncRNA SRA1 gene and PCOS susceptibility [16], while a study on the Iranian population found that the rs2067051G > A locus in the H19 gene raises PCOS risk in this group [17].

Rs5749201 is a polymorphic locus that has emerged prominently in disease research over recent years. Located within the gene region of the lncRNA TUG1, rs5749201 variation is significantly associated with the risk of developing several diseases, including systemic lupus erythematosus, lupus nephritis, and knee osteoarthritis [18, 19]. Furthermore, it has been confirmed to correlate with gene expression levels [19]. Taurine Upregulated Gene 1 (TUG1), an evolutionarily conserved lncRNA, is highly expressed in human ovaries, testes, and other tissues [20]. It participates in multiple biological processes and shows abnormal expression in metabolic disorders such as diabetes and hyperlipidemia [21, 22]. Latest findings indicate that high TUG1 expression in PCOS patients may trigger excessive follicle activation and growth [23]. Although TUG1’s rs5749201 locus is linked to systemic lupus erythematosus and osteoarthritis risk [18, 19], its connection to PCOS genetic susceptibility has not been documented.

In this study, we enrolled patients with PCOS and analyzed the distribution of the TUG1 rs5749201 locus in these subjects. Furthermore, a preliminary analysis was performed to investigate the association between the TUG1 rs5749201 locus and genetic susceptibility to PCOS.

## Materials and methods

### Study subjects

The study was conducted in accordance with the Declaration of Helsinki. A total of 210 patients with PCOS (all of Han Chinese ethnicity) were enrolled in this study, which was approved by the institutional ethics committee of Affiliated Beijing Chao-Yang Hospital, Capital Medical University. Prior to study initiation, written informed consent was obtained from all participants. All subjects underwent clinical and basic evaluations before enrollment, including assessments of reproductive and menstrual history, hirsutism, testosterone levels, and targeted ovarian ultrasound. Polycystic ovary syndrome (PCOS) was diagnosed in accordance with the 2004 Rotterdam Consensus Criteria [24], which mandate the presence of at least two out of three key features, with the concurrent exclusion of other endocrine disorders, namely: (1) oligomenorrhea or anovulation, defined as a menstrual cycle length exceeding 35 days or fewer than 8 spontaneous menstrual bleeding episodes per year; (2) clinical and/or biochemical hyperandrogenism, where biochemical hyperandrogenism was confirmed by a total testosterone level > 48.1 ng/dl in patients without clinical manifestations of hyperandrogenism, menstrual irregularities, or a history of hormonal medication use, and clinical hyperandrogenism was characterized by hirsutism with a modified Ferriman–Gallwey (mFG) score ≥ 6 [25]; and (3) polycystic ovarian morphology on transvaginal ultrasonography, identified by the presence of ≥ 12 antral follicles (2–9 mm in diameter) in at least one ovary and/or an ovarian volume ≥ 10 cm³. Additionally, the PCOS study population was categorized into four classic phenotypic subgroups according to established criteria [26], as defined below: Phenotype A (clinical/biochemical hyperandrogenism [HA], oligomenorrhea or anovulation [OA], and polycystic ovaries [PCO]); Phenotype B (HA and OA only); Phenotype C (HA and PCO only); and Phenotype D (OA and PCO only). Exclusion criteria were as follows: presence of Cushing’s syndrome, thyroid disorders, hyperprolactinemia, pituitary diseases, or androgen-secreting tumors; or use of hormones or steroids that induce insulin resistance at least 6 months prior to study onset. Notably, none of the included women had received any treatment at the time of data collection, and blood samples were collected during the follicular phase before treatment initiation.

In addition, 230 women with regular menstrual cycles and normal ovarian function confirmed through Ultrasound examinations were included as controls. Women with a history of cesarean section or pelvic surgery were excluded to avoid potential effects of ovarian manipulation. Additionally, participants who had experienced pregnancy, childbirth, or breastfeeding within the last year were excluded.

The statistical power was calculated using the G*power software version 3.1.9.4 (http://www.gpower.hhu.de/). This case–control study with the alpha error probability of 0.05 and the total sample size of two groups could provide a statistical power of 99.91% with a medium effect size of 0.35.

### Laboratory analysis

Following an overnight fast of at least 8 h, 10 mL of blood was drawn from each participant and transported to the laboratory for analysis of biochemical index (fasting blood glucose (FBS), homeostasis model assessment of insulin resistance (HOMA-IR), triglycerides (TG), total cholesterol (TC), low-density lipoprotein (LDL), high-density lipoprotein (HDL)) and hormonal indices (including free testosterone (Free-T), luteinizing hormone (LH), and follicle-stimulating hormone (FSH)).

### DNA extraction and genotyping method

Five milliliters of venous blood were collected from each participant, and genomic DNA was extracted from the whole blood samples using the MolPure^®^ Blood DNA Rapid Extraction Kit, strictly following the standard operating procedures provided by the manufacturer. For genotyping of the TUG1 rs5749201 polymorphism, the TaqMan™ SNP Genotyping Kit was employed. The total volume of the PCR reaction system was 20 µL, consisting of 10 µL of 2×TaqMan Universal PCR Master Mix, 1 µL of 20×TaqMan SNP Genotyping Assay (containing specific primers and probes), 1 µL of DNA sample (with a concentration of 10 ng/µL), and 8 µL of deionized water. The amplification reaction was performed on a real-time fluorescent quantitative PCR instrument, with the reaction program set as follows: initial denaturation at 95 °C for 10 min; followed by 40 cycles, each including denaturation at 95 °C for 15 s and annealing-extension at 60 °C for 1 min. To validate the accuracy, 10% of the samples were randomly selected for Sanger sequencing, and the concordance rate of genotyping results reached 100%.

### Quantitative real-time polymerase chain reaction (qRT-PCR)

Total RNA was isolated from serum samples with TRIzol reagent (Invitrogen, USA), following the protocol provided by the manufacturer. A Nanodrop 2000 spectrophotometer (Thermo Fisher Scientific, USA) was used to determine the concentration and purity of the extracted RNA. RNA samples with A260/A280 between 1.8–2.1 were used for cDNA synthesis by reverse transcription kit. cDNA samples were considered to have no genomic DNA contamination if the Ct value was equal to or greater than 35 for the corresponding no reverse transcription control RNA sample. For detecting the expression of TUG1, qRT-PCR was performed on an ABI 7900HT Real-Time PCR System (Applied Biosystems, USA), with the FastKing one-step RT-PCR kit (TIANGEN, Beijing) employed for the reaction. The primer sequence of lncRNA TUG1 (product size 166 bp) is F: 5’-TAGCAGTTCCCCAATCCT TG-3’ and R: 5’-CACAAA TTCCCATCATCCC-3’. The primer sequence of GAPDH (product size 177 bp) is 5’-ATGACATCAAGAAGGTGGTG-3’ and 5’-CATACCAGGAAATGAGCTTG-3’. PCR reaction conditions: initial denaturation at 94˚C for 2 min, followed by 40 cycles of 94˚C for 20 s, 60˚C for 34 s. Each qRT-PCR was performed in three technical replicates. No outliers were removed, as all replicate Ct values fell within the acceptable range. The relative expression of TUG1 was normalized against the housekeeping gene GAPDH [27], and its value was calculated using the 2^(−ΔΔCt)^ method. Primer amplification efficiency (PAE) of GAPDH and TUG1 was 93.7% and 92.9% respectively.

### Statistical analysis

All statistical analyses were conducted with SPSS software (Version 21). The Hardy-Weinberg equilibrium (HWE) was verified through a chi-square test to verify the representativeness of the study subjects. For variable representation, all quantitative variables were presented as mean ± standard deviation (SD), while qualitative variables were expressed as percentages. To compare demographic characteristics between the two groups, statistical methods were selected based on variable types: the chi-square test was used for categorical variables, and Student’s t-test was applied for continuous variables. One-way ANOVA post-Bonferroni multiple comparisons test was applied for comparisons among more than three groups. Pearson’s correlation analysis was performed to evaluate the association between continuous variables. The relationship between gene polymorphism and PCOS risk was analyzed via multivariate logistic regression, with adjustments made for other relevant clinical indicators. Statistical significance was defined as a two-sided *P*-value < 0.05.

## Results

### Baseline characteristics of the study groups

In this study, 210 patients with PCOS and 230 healthy volunteers were recruited as the case and control groups, respectively. Baseline characteristics analysis (Table 1) revealed no significant between-group differences in the distributions of age and BMI. However, compared to the control group, the PCOS group exhibited significantly higher levels of blood lipid and glucose-related parameters, with the exception of TC, which did not differ significantly. Additionally, patients with PCOS had markedly elevated LH levels and LH/ FSH ratios. While FSH and estradiol levels showed an increasing trend in the PCOS group, these differences did not reach statistical significance.

**Table 1:** Demographical and clinical characteristics of PCOS patients and controls

Characteristics	Control (*n* = 230)	PCOS (*n* = 210)	*P* value
Age, year	28.99 ± 4.06	28.62 ± 3.88	0.333
BMI, kg/m^2^	22.94 ± 3.95	23.65 ± 5.22	0.110
FBS, mmol/L	5.04 ± 0.71	5.66 ± 0.94	< 0.001
HOMA-IR	1.99 ± 0.56	2.55 ± 0.93	< 0.001
TC, mmol/L	4.53 ± 0.59	4.64 ± 0.78	0.078
TG, mmol/L	1.23 ± 0.46	1.70 ± 0.66	< 0.001
LDL, mmol/L	2.26 ± 0.52	2.79 ± 0.82	< 0.001
HDL, mmol/L	1.52 ± 0.51	1.31 ± 0.51	< 0.001
LH, mIU/mL	7.11 ± 1.00	10.11 ± 2.46	< 0.001
FSH, mIU/mL	5.57 ± 0.68	5.64 ± 0.92	0.316
LH/FSH	1.30 ± 0.25	1.84 ± 0.54	< 0.001
Estradiol level, pmol/L	80.72 ± 10.03	82.56 ± 11.70	0.079
Phenotype, n			-
A	-	74	
B	-	21	
C	-	32	
B	-	83	

The Student’s t-test was applied for difference comparison between groups

*PCOS* Polycystic ovary syndrome, *BMI* Body mass index, *FBS* Fasting blood sugar, *HOMA-IR* Homeostasis model assessment of insulin resistance, *TC* Total cholesterol, *TG* Triglyceride, *LDL* Low density lipoprotein, *HDL* High density lipoprotein, *LH* Luteinizing hormone, *FSH* Follicle-stimulating hormone

### Genetic association of TUG1 rs5749201 locus and PCOS

Table 2 summarizes the genotype distribution of the TUG1 rs5749201 locus. The genotype frequency distribution in the control group did not deviate from HWE (*P* > 0.05), with no evidence of population stratification. In terms of genotype frequency comparison, the PCOS group showed a significantly higher proportion of AA genotype carriers and a significantly lower proportion of TT genotype carriers relative to the control. Further risk association analysis revealed that compared with carriers of the AA genotype, people carrying the TT genotype had significantly lower risk of developing PCOS, suggesting that the A allele might be a pathogenic allele related to PCOS. Furthermore, through hierarchical analyses of the dominant model and the recessive model, both models verified the genetic association between the TUG1 rs5749201 locus and the onset of PCOS. In the dominant genetic model, we observed that carriers of at least one mutant T allele exhibited a markedly reduced risk (OR = 0.510, 95%CI = 0.300-0.866, *P* = 0.012) of developing PCOS compared with individuals homozygous for the wild-type A allele. Under the recessive genetic model, the homozygous mutant TT genotype was still identified as a risk factor for PCOS (OR = 0.594, 95%CI = 0.395–0.893, *P* = 0.012).

**Table 2:** Genotype and allele distributions of TUG1 rs5749201 polymorphism in PCOS patients

Genetic models	Control, % (*n* = 230)	PCOS, % (*n* = 210)	χ 2	OR (95% CI)	*P*
AA	26 (11.30)	42 (20.00)	-	1	-
AT	118 (51.30)	113 (53.81)	3.473	0.593 (0.341–1.031)	0.062
TT	86 (37.39)	55 (26.19)	9.553	0.396 (0.218–0.718)	0.002
Dominant					
AA	26 (11.30)	42 (20.00)	-	1	-
AT/TT	204 (88.70)	168 (80.00)	6.353	0.510 (0.300-0.866)	0.012
Recessive					
AA/AT	144 (62.61)	155 (73.80)	-	1	-
TT	86 (37.39)	55 (26.19)	6.324	0.594 (0.395–0.893)	0.012
Alleles					
A	170 (36.96)	197 (46.90)	-	1	-
T	290 (63.04)	223 (53.10)	8.937	0.664 (0.507–0.869)	0.003
*P* ^HWE^	0.126				

The chi-square test was used for difference comparison between two groups

*HWE* Hardy-Weinberg Equilibrium, *OR* Odd ratio, *CI* Confidence interval

To further confirm the association between the TUG1 rs5749201 locus and PCOS, additional clinically relevant indicators known to be linked with PCOS were incorporated into a multivariate logistic regression model (Table 3). To mitigate multicollinearity issues, six core indicators were selected based on variance inflation factors (VIF; supplementary Table 1) and used to construct a simplified model. Its fitting performance was compared with that of the initial model containing all indicators. Results showed that the simplified model exhibited significantly lower AIC (557 vs. 593) and BIC (586 vs. 615) values, with improved pseudo R-squared (0.138 vs. 0.057). Furthermore, the Hosmer-Lemeshow tests for both models met the criteria for good fit (*P* > 0.05). This indicated that after variable selection via VIF, the simplified model not only reduced multicollinearity but also demonstrated superior fit and explanatory power compared to the initial model. Analyses revealed that following adjustment for these clinical covariates, the TUG1 rs5749201 locus remained independently associated with PCOS in a statistically significant manner (OR = 0.427, 95%CI: 0.242–0.753; *P* = 0.003), supporting the notion that this locus may serve as an independent factor implicated in PCOS pathogenesis.

**Table 3:** Multivariate logistic regression analysis of factors related to the onset of PCOS

Characteristics	OR	95% CI	*P* value
HOMA-IR (> 2.26)	1.642	1.074–2.510	0.022
LDL (> 2.51 mmol/L)	1.723	1.120–2.652	0.013
LH/FSH (> 1.56)	4.306	2.770–6.693	< 0.001
Rs5749201 (AT/TT)	0.427	0.242–0.753	0.003
Age (> 28)	0.937	0.622–1.412	0.755
BMI (> 23.5 kg/m2)	1.181	0.787–1.773	0.422

The multivariate logistic regression analysis was performed to independent relationship between various variables and PCOS

*PCOS* Polycystic ovary syndrome, *BMI* Body mass index, *FBS* Fasting blood sugar, *HOMA-IR* Homeostasis model assessment of insulin resistance, *TC* Total cholesterol, *TG* Triglyceride, *LDL* Low density lipoprotein, *HDL* High density lipoprotein, *LH* Luteinizing hormone, *FSH* Follicle-stimulating hormone, *OR* Odd ratio, *CI* Confidence interval.

### Association of clinical characteristics with genotypic frequencies of TUG1 rs5749201 in PCOS patients

PCOS patients were stratified into distinct subgroups according to their TUG1 rs5749201 genotypes, and comparisons of clinical baseline characteristics were conducted across these subgroups. As presented in Table 4, carriers of the AA genotype exhibited significantly elevated levels of LDL, TG, and FBS relative to AT/TT genotype carriers, with statistically significant differences observed between groups (*P* < 0.05). In contrast, no significant variations in the distribution of other clinical indicators were detected among carriers of different genotypes. These findings suggest that the TUG1 rs5749201 locus may be involved in regulating lipid and glucose metabolism in patients with PCOS.

**Table 4:** Association of clinical characteristics with genotypic frequencies of TUG1 rs5749201 polymorphism in PCOS patients

Characteristics	AA (*n* = 42)	AT/TT (*n* = 168)	*P* value
Age, year	27.74 ± 3.90	28.85 ± 3.85	0.098
BMI, kg/m^2^	23.51 ± 4.33	23.69 ± 5.43	0.844
FBS, mmol/L	5.93 ± 0.82	5.60 ± 0.96	0.037
HOMA-IR	2.65 ± 0.75	2.53 ± 0.98	0.459
TC, mmol/L	4.65 ± 0.82	4.64 ± 0.78	0.976
TG, mmol/L	1.93 ± 0.59	1.64 ± 0.67	0.012
LDL, mmol/L	3.08 ± 0.73	2.71 ± 0.82	0.009
HDL, mmol/L	1.21 ± 0.44	1.34 ± 0.52	0.130
LH, mIU/mL	10.68 ± 2.03	9.96 ± 2.54	0.090
FSH, mIU/mL	5.60 ± 0.88	5.66 ± 0.93	0.721
LH/FSH	1.91 ± 0.62	1.82 ± 0.51	0.312
Estradiol level, pmol/L	83.19 ± 12.66	82.40 ± 11.48	0.698

*PCOS* Polycystic ovary syndrome, *BMI* Body mass index, *FBS* Fasting blood sugar, *HOMA-IR* Homeostasis model assessment of insulin resistance, *TC* Total cholesterol, *TG* Triglyceride, *LDL* Low density lipoprotein, *HDL* High density lipoprotein, *LH* Luteinizing hormone, *FSH* Follicle-stimulating hormone

### Serum TUG1 levels in PCOS patients and its association with metabolic/hormonal parameters

**Fig. 1:**
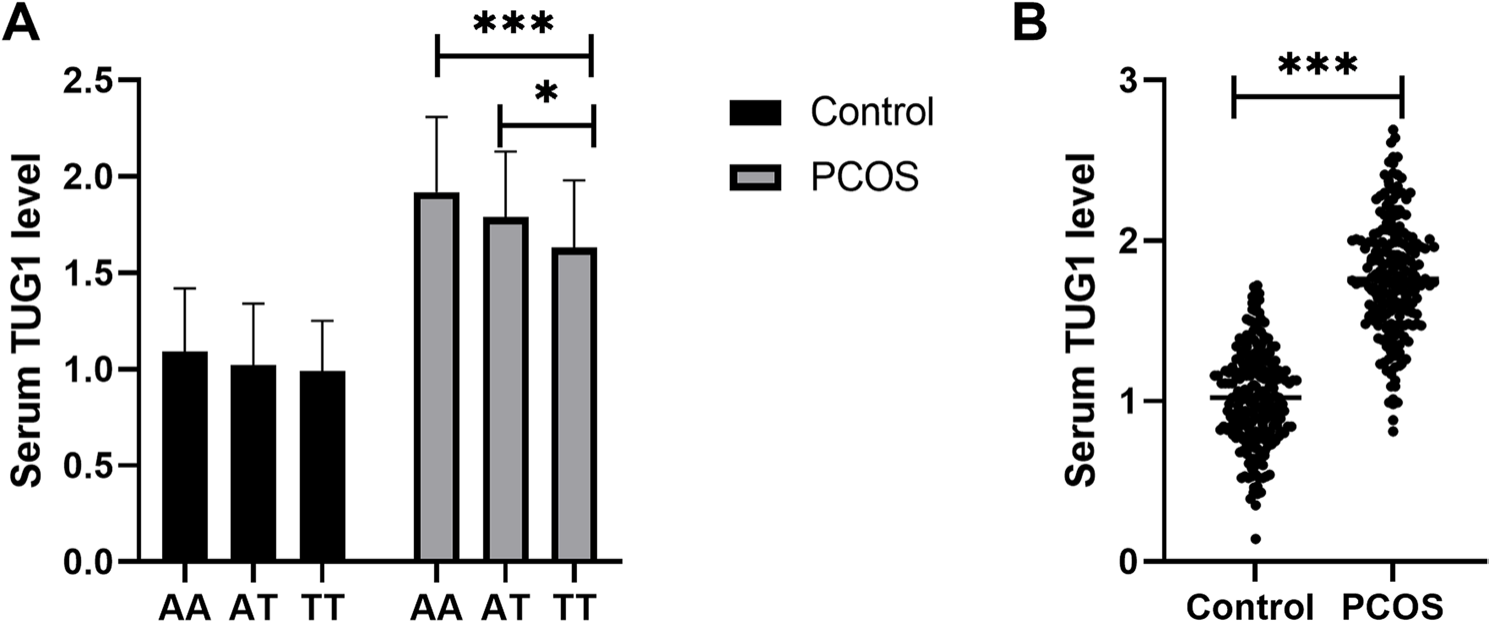
Serum TUG1 levels in PCOS patients. **A**. Serum TUG1 levels in individuals with different rs5749201 genotypes. **B**. Serum TUG1 levels in PCOS patients and controls. One-way ANOVA post-Bonferroni multiple comparisons test was applied for comparisons among different groups. *, *P* < 0.05; ***, *P* < 0.001

Patients with PCOS were categorized into distinct subgroups based on their TUG1 rs5749201 genotypes, and serum TUG1 levels were compared across these subgroups. As illustrated in Fig. 1A, no significant differences in serum TUG1 levels were observed among carriers of different genotypes in the control group. In contrast, within the PCOS group, carriers of the rs5749201 AA genotype exhibited the highest serum TUG1 levels, whereas those with the TT genotype had the lowest, with these intergroup differences reaching statistical significance (*P* < 0.05). Additionally, a further comparison of serum TUG1 levels between the control and PCOS groups revealed a significant elevation in TUG1 levels among PCOS patients (Fig. 1B). The results demonstrated that patients with PCOS had significantly elevated TUG1 levels, with carriers of the rs5749201 AA genotype showing the highest serum TUG1 levels among this patient group. Moreover, its association with key metabolic/hormonal parameters in PCOS patients was further evaluated (Table 5). The data indicated that serum TUG1 was positively associated with FBS (*r* = 0.345, *P* < 0.001), TG (*r* = 0.655, *P* < 0.001) and LDL (*r* = 0.674, *P* < 0.001).

**Table 5:** Association of TUG1 expression with key metabolic/hormonal parameters in PCOS patients

Characteristics	*r*	*P* value
FBS, mmol/L	0.345	< 0.001
HOMA-IR	0.125	0.070
TC, mmol/L	0.079	0.252
TG, mmol/L	0.655	< 0.001
LDL, mmol/L	0.674	< 0.001
HDL, mmol/L	0.066	0.341
LH/FSH	0.096	0.167

Association was evaluated via Pearson’s correlation analysis

*PCOS* Polycystic ovary syndrome, *FBS* Fasting blood sugar, *HOMA-IR* Homeostasis model assessment of insulin resistance, *TC* Total cholesterol, *TG* Triglyceride, *LDL* Low density lipoprotein, *HDL* High density lipoprotein, *LH* Luteinizing hormone, *FSH* Follicle-stimulating hormone

## Discussion

LncRNA TUG1 serves as a crucial regulator of cellular processes, including proliferation, apoptosis, differentiation, and metabolism [28, 29]. Notably, rs5749201, a prevalent SNP located in the TUG1 gene, has been validated to modulate TUG1 expression and is associated with the risk of developing systemic lupus erythematosus and knee osteoarthritis [18, 19]. Building on this existing body of evidence, the findings of the present study further augment the understanding of TUG1-related genetic mechanisms. Specifically, the current findings highlighted a clear genetic correlation between the TUG1 rs5749201 locus and PCOS, offering a novel avenue for investigating the genetic underpinnings of PCOS. It is known that large-scale PCOS GWAS have mainly focused on common variants in European or multi-ethnic populations, with inadequate coverage of variants in lncRNA intronic regulatory regions. Our candidate gene analysis of TUG1 functional variants in East Asian populations thus fills this critical gap, serving as a vital complementary approach to decipher the genetic etiology of PCOS. This study identifies a TUG1-lncRNA SNP as the first genome-wide significant susceptibility variant for PCOS in Han Chinese, addressing a long-standing gap in lncRNA research and minimizing population stratification. The risk allele is markedly enriched in affected Han women and shows pronounced East-Asian-specific elevation compared with the European/African cohort [30], offering a genetic clue to ethnic disparity in PCOS prevalence. The SNP defines a novel Han-Chinese PCOS locus for precision prevention; cross-ethnic replication and mechanistic follow-up are warranted.

Metabolic disorders, such as insulin resistance and dyslipidemia, are one of the core pathological features of PCOS [31–33]. This study found that individuals carrying the AA genotype at the TUG1 rs5749201 locus had significantly higher levels of LDL, TG, and FBS. This result tentatively links the locus to PCOS metabolic status, but mechanistic claims are unsupported by functional evidence. This finding also provided a preliminary reference for the “classification-based management” of PCOS, but requires cautious interpretation. For PCOS patients carrying the rs5749201 AA genotype, in clinical practice, more active metabolic intervention measures, such as personalized dietary regulation, regular exercise guidance, etc., might be required to effectively reduce their long-term risks of developing cardiovascular diseases, type 2 diabetes, and other complications. Nevertheless, these conclusions remain to be corroborated by large-sample investigations and rigorous clinical validation trials.

SNPs can affect an individual’s susceptibility to diseases by regulating the expression level of genes [34]. The results of this study showed that carriers of the AA genotype at the TUG1 rs5749201 locus had the highest serum TUG1 levels, and PCOS patients generally exhibited a high expression of TUG1. Previous literature has confirmed that TUG1 is significantly upregulated in PCOS patients, and this high expression is closely related to excessive activation and abnormal growth of follicles, suggesting that TUG1 has the potential to serve as a diagnostic biomarker for PCOS [23]. This is highly consistent with the results of this study. Based on the above findings, we proposed the following mechanism hypothesis that the abnormal high expression of TUG1 was one of the important molecular mechanisms of PCOS onset, and the rs5749201 AA genotype may enhance the expression level of TUG1 by strengthening its regulatory effect on follicle development, ultimately increasing the risk of individuals developing PCOS. Although TUG1 is mainly located in the cell nucleus, under pathological conditions, it can enter the bloodstream through the cross-cellular communication pathway mediated by extracellular vesicles, such as exosomes. It is reported that TUG1 can be loaded into EVs secreted by endothelial progenitor cells (EPCs) [35]. The TUG1 detected in serum is likely to be a nuclear component released from damaged cells or specific secretory cells. However, the source of this needs further research to confirm.

However, this study still has several limitations that need to be improved and expanded in subsequent research. Firstly, PCOS is a typical polygenic disorder, yet the synergistic interactions among the multiple susceptibility genes underlying its pathogenesis have not been fully elucidated. Secondly, the exact genetic mechanisms governing PCOS remain incompletely understood. Furthermore, PCOS exhibits marked clinical phenotypic heterogeneity, which not only adds to the complexity of dissecting its genetic underpinnings but also presents a major challenge for elucidating the genotype-phenotype correlations. In subsequent studies, we will integrate the TUG1 rs5749201 locus with relevant environmental variables and clinical background information, with the aim of accurately identifying and validating key genes that exert core regulatory functions in the pathogenesis of PCOS.

By employing multi-dimensional analytical approaches, this study confirmed the genetic link between the TUG1 rs5749201 locus and PCOS. It also shed preliminary light on the involvement of the rs5749201 locus in regulating TUG1 expression. Collectively, the genetic link between TUG1 gene polymorphisms and PCOS susceptibility offers a genetic basis for individualized nursing strategies, enabling targeted early lifestyle guidance and risk stratification in women with a family history of PCOS. Nevertheless, these conclusions remain to be corroborated by large-sample investigations and rigorous clinical validation trials.

## Supplementary Information

Supplementary Material 1.

## Data Availability

The datasets generated during and/or analysed during the current study are available from the corresponding author on reasonable request.

## References

[1] Williams RM, Ong KK, Dunger DB. Polycystic ovarian syndrome during puberty and adolescence. Mol Cell Endocrinol. 2013;373(1–2):61–7. 23384539 10.1016/j.mce.2013.01.005

[2] Shannon M, Wang Y. Polycystic ovary syndrome: a common but often unrecognized condition. J Midwifery Women’s Health. 2012;57(3):221–30. 22594862 10.1111/j.1542-2011.2012.00161.x

[3] Ali AT, Al-Ani O, Al-Ani F, Guidozzi F. Polycystic ovary syndrome and metabolic disorders: A review of the literature. Afr J Reprod Health. 2022;26(8):89–99. 37585035 10.29063/ajrh2022/v26i8.9

[4] Mykhalchenko K, Lizneva D, Trofimova T, Walker W, Suturina L, Diamond MP, et al. Genetics of polycystic ovary syndrome. Expert Rev Mol Diagn. 2017;17(7):723–33. 28602111 10.1080/14737159.2017.1340833

[5] de Alencar JB, Alves HV, Elpidio LN, Visentainer JE, Sell AM. Polymorphisms of cytokine genes and polycystic ovary syndrome: A review. Metab Syndr Relat Disord. 2016;14(10):468–74. 27809669 10.1089/met.2016.0101

[6] Rezgoun ML, El Khour D, Bendaoud H, Chellat D. CYP17A1 (rs74357) polymorphism and polycystic ovary syndrome risk: a systemic review and meta-analysis. Acta Biomed. 2023;94(4):e2023167. 37539608 10.23750/abm.v94i4.14229 PMC10440780

[7] Zhang C, Yu J. FTO gene polymorphism and susceptibility to polycystic ovary syndrome: A meta-analysis. J Obstet Gynaecol Res. 2024;50(9):1703–12. 39143730 10.1111/jog.16047

[8] Albahlol IA, Neamatallah M, Serria MS, El-Gilany AH, Setate YA, Alkasaby NM, et al. Vitamin D receptor gene polymorphism and polycystic ovary syndrome susceptibility. BMC Med Genom. 2023;16(1):108. 10.1186/s12920-023-01541-8 PMC10197246 37202765

[9] Heidarzadehpilehrood R, Hamid HA, Pirhoushiaran M. Vitamin D receptor (VDR) gene polymorphisms and risk for polycystic ovary syndrome and infertility: an updated systematic review and meta-analysis. Metabolism Open. 2025;25:100343. 39866289 10.1016/j.metop.2024.100343 PMC11764755

[10] Heidarzadehpilehrood R, Pirhoushiaran M, Osman MB, Ling KH, Hamid HA. Identifying genetic profiles in peripheral blood mononuclear cells in women with polycystic ovary syndrome: an observational Case-Control study. Arch Med Res. 2025;56(3):103129. 39647252 10.1016/j.arcmed.2024.103129

[11] Herman AB, Tsitsipatis D, Gorospe M. Integrated LncRNA function upon genomic and epigenomic regulation. Mol Cell. 2022;82(12):2252–66. 35714586 10.1016/j.molcel.2022.05.027 PMC9219586

[12] Heidarzadehpilehrood R, Pirhoushiaran M, Binti Osman M, Ling KH, Abdul Hamid H. Unveiling key biomarkers and therapeutic drugs in polycystic ovary syndrome (PCOS) through pathway enrichment analysis and hub Gene-miRNA networks. Iran J Pharm Research: IJPR. 2023;22(1):e139985. 38444712 10.5812/ijpr-139985 PMC10912876

[13] Heidarzadehpilehrood R, Pirhoushiaran M, Binti Osman M, Abdul Hamid H, Ling KH. Weighted gene Co-Expression network analysis (WGCNA) discovered novel long Non-Coding RNAs for polycystic ovary syndrome. Biomedicines. 2023;11(2):518. 10.3390/biomedicines11020518 PMC9953234 36831054

[14] Wang K, Cheng Y, Ren Y, Xiu H, Meng W, Guo T, et al. LncRNA S100PBP promotes proliferation and steroid hormone synthesis of granulosa cells by sponging MiR-2285bc-BMPR2 in bovine†. Biol Reprod. 2024;111(1):92–109. 38412119 10.1093/biolre/ioae033

[15] Bai L, Gong J, Guo Y, Li Y, Huang H, Liu X. Construction of a CeRNA network in polycystic ovary syndrome (PCOS) driven by Exosomal LncRNA. Front Genet. 2022;13:979924. 36406137 10.3389/fgene.2022.979924 PMC9672461

[16] Tan J, Hao X, Zhao T, Ying J, Li T, Cheng L. Association between long-chain non-coding RNA SRA1 gene single-nucleotide polymorphism and polycystic ovary syndrome susceptibility. J Assist Reprod Genet. 2020;37(10):2513–23. 32783135 10.1007/s10815-020-01922-3 PMC7550517

[17] Ghasemi M, Heidari Nia M, Hashemi M, Keikha N, Fazeli K, Taji O, et al. An association study of polymorphisms in the H19 imprinted gene in an Iranian population with the risk of polycystic ovary syndrome. Biol Reprod. 2020;103(5):978–85. 32720692 10.1093/biolre/ioaa131

[18] Tawfeek GA, Kasem H, Abdallah EA, Almulhim M, Almulhim A, Albarqi M et al. Long Non-Coding RNA TUG1 gene polymorphism and TUG1 expression level as molecular biomarkers of systemic lupus erythematosus and lupus nephritis. Non-coding RNA. 2023;9(5):56. 10.3390/ncrna9050056 PMC10514853 37736902

[19] Duan J, Shen T, Dong H, Han S, Li G. Association of the expression levels of Long-Chain noncoding RNA TUG1 and its gene polymorphisms with knee osteoarthritis. Genetic Test Mol Biomarkers. 2021;25(2):102–10. 10.1089/gtmb.2020.0208 33596137

[20] Chen T, Lu J, Fan Q. LncRNA TUG1 and kidney diseases. BMC Nephrol. 2025;26(1):139. 40108517 10.1186/s12882-025-04047-w PMC11924614

[21] Ageeli Hakami M. Diabetes and diabetic associative diseases: an overview of epigenetic regulations of TUG1. Saudi J Biol Sci. 2024;31(5):103976. 38510528 10.1016/j.sjbs.2024.103976 PMC10951089

[22] Zhang L, Cheng H, Yue Y, Li S, Zhang D, He R. TUG1 knockdown ameliorates atherosclerosis via up-regulating the expression of miR-133a target gene FGF1. Cardiovasc Pathology: Official J Soc Cardiovasc Pathol. 2018;33:6–15. 10.1016/j.carpath.2017.11.004 29268138

[23] Li Y, Zhang J, Liu YD, Zhou XY, Chen X, Zhe J, et al. Long non-coding RNA TUG1 and its molecular mechanisms in polycystic ovary syndrome. RNA Biol. 2020;17(12):1798–810. 32559120 10.1080/15476286.2020.1783850 PMC7714456

[24] Rotterdam ESHRE/ASRM-Sponsored PCOS Consensus Workshop Group. Revised 2003 consensus on diagnostic criteria and long-term health risks related to polycystic ovary syndrome. Fertil Steril. 2004;81(1):19–25. 10.1016/j.fertnstert.2003.10.004 14711538

[25] Chen ZJ, Shi Y, Sun Y, Zhang B, Liang X, Cao Y, et al. Fresh versus frozen embryos for infertility in the polycystic ovary syndrome. N Engl J Med. 2016;375(6):523–33. 27509101 10.1056/NEJMoa1513873

[26] Norman RJ, Dewailly D, Legro RS, Hickey TE. Polycystic ovary syndrome. Lancet (London England). 2007;370(9588):685–97. 17720020 10.1016/S0140-6736(07)61345-2

[27] Karataylı E, Altunoğlu Y, Karataylı SC, Yurdaydın C, Bozdayı AM. Free Circulating nucleic acids in plasma and serum as a novel approach to the use of internal controls in real time PCR based detection. J Virol Methods. 2014;207:133–7. 25034126 10.1016/j.jviromet.2014.07.008

[28] He C, Li Z, Yu W, Luo R, Zhou J, He J, et al. LncRNA TUG1 mediates microglial inflammatory activation by regulating glucose metabolic reprogramming. Sci Rep. 2024;14(1):12143. 38802677 10.1038/s41598-024-62966-4 PMC11130314

[29] López-Noriega L, Rutter GA. Long Non-Coding RNAs as key modulators of pancreatic β-Cell mass and function. Front Endocrinol. 2020;11:610213. 10.3389/fendo.2020.610213 PMC7897662 33628198

[30] Zhao H, Xu Y, Xue B, Zhao S, Zhang M, Wu XK, et al. Multi-ancestry genome-wide association analyses of polycystic ovary syndrome. Nat Genet. 2025;57(11):2669–81. 41188533 10.1038/s41588-025-02393-x

[31] Siddiqui S, Mateen S, Ahmad R, Moin S. A brief insight into the etiology, genetics, and immunology of polycystic ovarian syndrome (PCOS). J Assist Reprod Genet. 2022;39(11):2439–73. 36190593 10.1007/s10815-022-02625-7 PMC9723082

[32] Di Lorenzo M, Cacciapuoti N, Lonardo MS, Nasti G, Gautiero C, Belfiore A, et al. Pathophysiology and nutritional approaches in polycystic ovary syndrome (PCOS): A comprehensive review. Curr Nutr Rep. 2023;12(3):527–44. 37213054 10.1007/s13668-023-00479-8 PMC10444658

[33] Abraham Gnanadass S, Divakar Prabhu Y, Valsala Gopalakrishnan A. Association of metabolic and inflammatory markers with polycystic ovarian syndrome (PCOS): an update. Arch Gynecol Obstet. 2021;303(3):631–43. 33439300 10.1007/s00404-020-05951-2

[34] Li HT, Yuan SX, Wu JS, Zhang XZ, Liu Y, Sun X. Molecular subtyping of mild cognitive impairment based on genetic polymorphism and gene expression. J Prev Alzheimer’s Disease. 2021;8(2):224–33. 33569571 10.14283/jpad.2020.65

[35] Ma W, Zhang W, Cui B, Gao J, Liu Q, Yao M, et al. Functional delivery of LncRNA TUG1 by endothelial progenitor cells derived extracellular vesicles confers anti-inflammatory macrophage polarization in sepsis via impairing miR-9-5p-targeted SIRT1 Inhibition. Cell Death Dis. 2021;12(11):1056. 34743197 10.1038/s41419-021-04117-5 PMC8572288

