# Supplementary material for: Genetic polymorphism of long non-coding RNA TUG1 and susceptibility to polycystic ovary syndrome: a case-control study
Source: Hereditas. 2026 Jan 22;163:27. doi: 10.1186/s41065-026-00638-1 (PMC12911283; doi:10.1186/s41065-026-00638-1)
Supplement: Supplementary file 1 — Supplementary Material 1. [file 41065_2026_638_MOESM1_ESM.docx]

**Supplementary Table 1** Independent variable multicollinearity test (VIF value)

| Variables | VIF |
| --- | --- |
| HOMA-IR | 1.014 |
| LDL | 1.004 |
| LH/FSH | 1.013 |
| Rs5749201 | 1.007 |
| Age | 1.022 |
| BMI | 1.010 |

Note: VIF, Variance Inflation Factor; BMI, body mass index; HOMA-IR, homeostasis model assessment of insulin resistance; LDL, low density lipoprotein; LH, luteinizing hormone; FSH, follicle-stimulating hormone.
